# Surgical Management of Flexion Type Supracondylar Humeral Fracture With Ulnar Nerve Injury - A Report of a Rare Case

**DOI:** 10.7759/cureus.26433

**Published:** 2022-06-29

**Authors:** Kishore Vellingiri, Meenakshi S Andra Suryanarayana, Balasaranaya Sambathkumar, Hariprasad Seenappa

**Affiliations:** 1 Hand Surgery, Kasturba Medical College of Manipal, Manipal, IND; 2 Orthopedics, Sri Devaraj Urs Academy of Higher Education and Research, Kolar, IND; 3 Biochemistry, Peelamedu Samanaidu Govindasamy Institute of Medical Sciences and Research, Coimbatore, IND; 4 Medicine, Coimbatore Medical College, Coimbatore, IND; 5 Orthopedics, Sri Devaraj Urs Academy of Higher Eduacation and Research, Kolar, IND

**Keywords:** ulnar clawing, kirschner wire, ulnar nerve injury, flexion type, supra-condylar fracture humerus

## Abstract

Supracondylar humerus fractures in children account for up to two-thirds of pediatric elbow injuries that require hospitalization. Supra-condylar fractures usually occur due to falls from height, from sports, or through acts of leisure. The estimation of their incidence is about 177.3 per 100000. Here, we report a successfully managed case of flexion-type supra-condylar fracture of the humerus with ulnar nerve injury without any complications.

## Introduction

Supra-condylar fractures of the humerus account for 55% to 80% of total elbow fractures [1]. These fractures in children account for up to two-thirds of pediatric elbow injuries that require hospitalization [1]. Supra-condylar fractures usually occur due to falls from height, from sports, or from acts of leisure. The estimation of their incidence is about 177.3 per 100000 [2]. Flexion-type fractures represent about 1% to 3% of cases. The most common cause of supra-condylar fracture is usually direct trauma to the flexed elbow [3]. We report the successful management of delayed presentation of flexion-type supracondylar fracture of the humerus with ulnar nerve injury without any complications.

This case study was presented as a poster at the 64th Annual Conference of the Indian Orthopedic Association (IOACON 2019) held between the 19th and 24th of November 2019 in Kolkata, India. The abstract of this article is published in the online supplement of the conference journal.

## Case presentation

**Figure 1:**
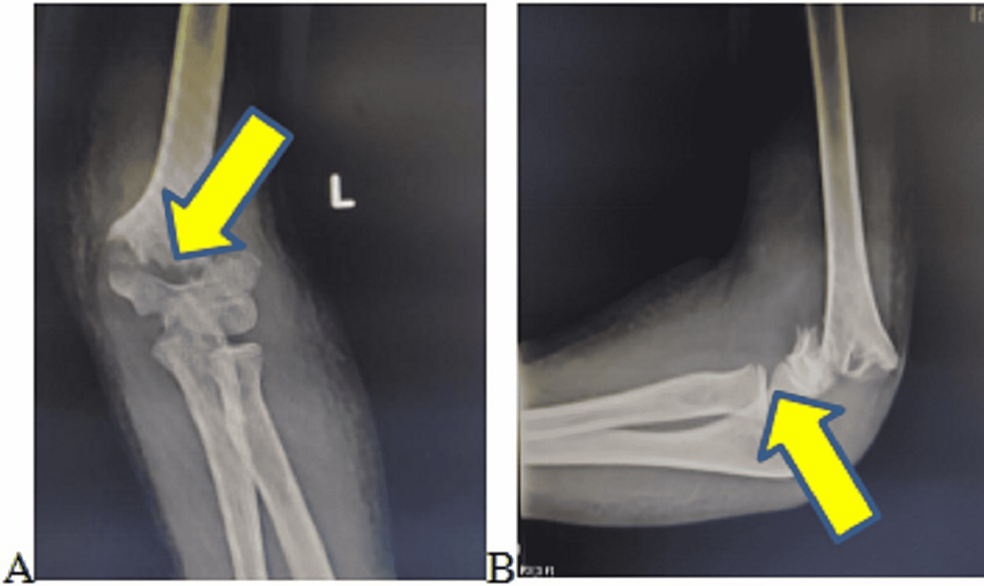
Plain radiograph of left elbow (A) antero-posterior view and (B) lateral view showing left supracondylar fracture with gross rotational anterolateral displacement

**Figure 2:**
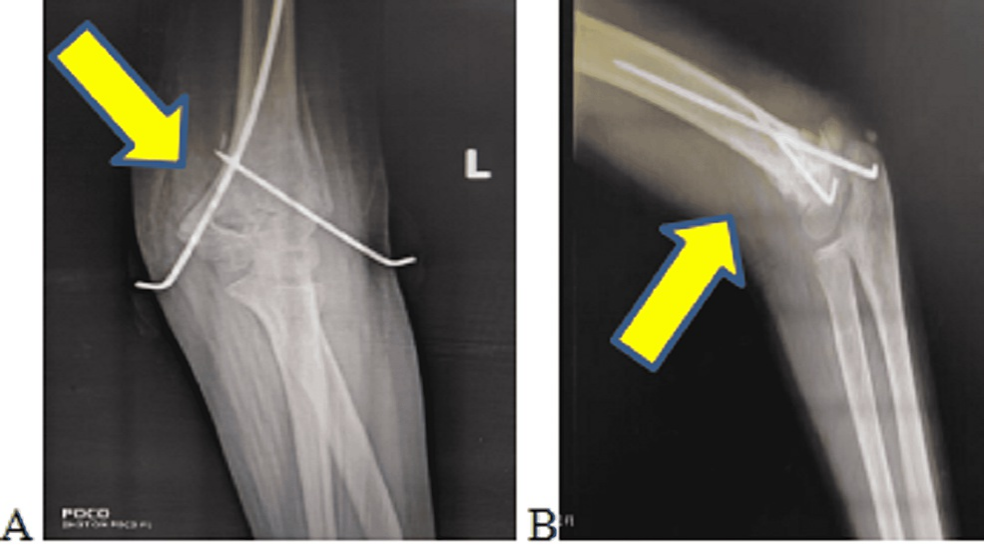
Plain radiographs of the left elbow (A) antero-posterior view and (B) lateral view showing Kirschner wire fixation for left supracondylar fracture with gross rotational anterolateral displacement

A 10-year-old female patient was brought to our tertiary care center at Kolar, Karnataka, with an alleged history of a fall when she sustained an injury over her left upper limb three weeks following the initial injury. The patient presented with swelling and pain over her left elbow with the inability to move her left little finger since the fall. She had no history of injuries elsewhere in her body. On examination, the left elbow joint was tender, grossly swollen, and in a slightly extended position. The three-point relation could not be assessed due to the swelling. The range of motion at the left elbow joint was painful and restricted. Sensation was lost in her left little finger, and extension of all other fingers was present. Brachial artery pulsation was palpable, and no vascular deficits were noticed. Anti-edema measures were taken through limb elevation over the pillow, above elbow plaster of Paris slab application in extension, and medications. Analgesics and prophylactic antibiotics were started, and the dosage was as per the weight of the child. The patient was sent for a plain radiograph of the left elbow joint. Radiographs showed left supracondylar fracture with gross rotational anterolateral displacement, as shown in Figure 1. As a trial, closed manipulation was attempted, and it failed as expected. After obtaining proper written, informed consent from her guardian, the patient underwent open reduction and internal fixation with two criss-cross Kirschner wire fixations, as shown in Figure 2, and above-the-elbow slab immobilization for four weeks.

Hospital course

**Figure 3:**
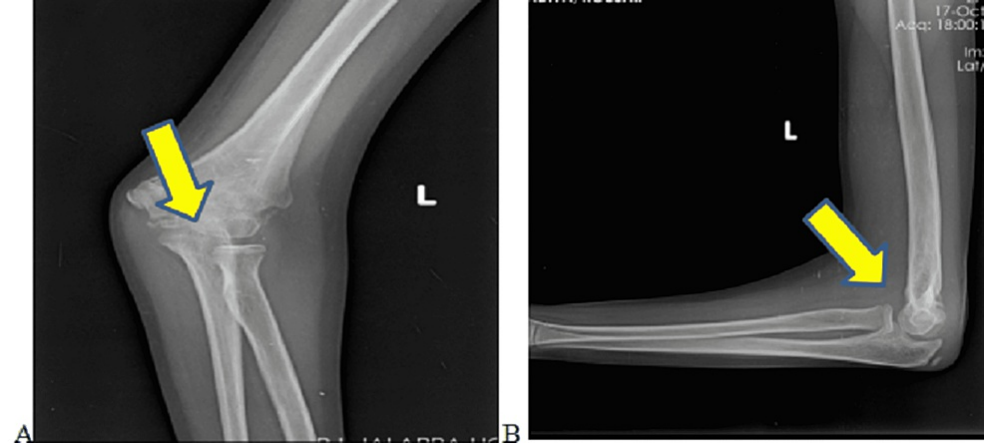
Plain radiographs of the left elbow (A) antero-posterior and (B) lateral views at three months follow-up

**Figure 4:**
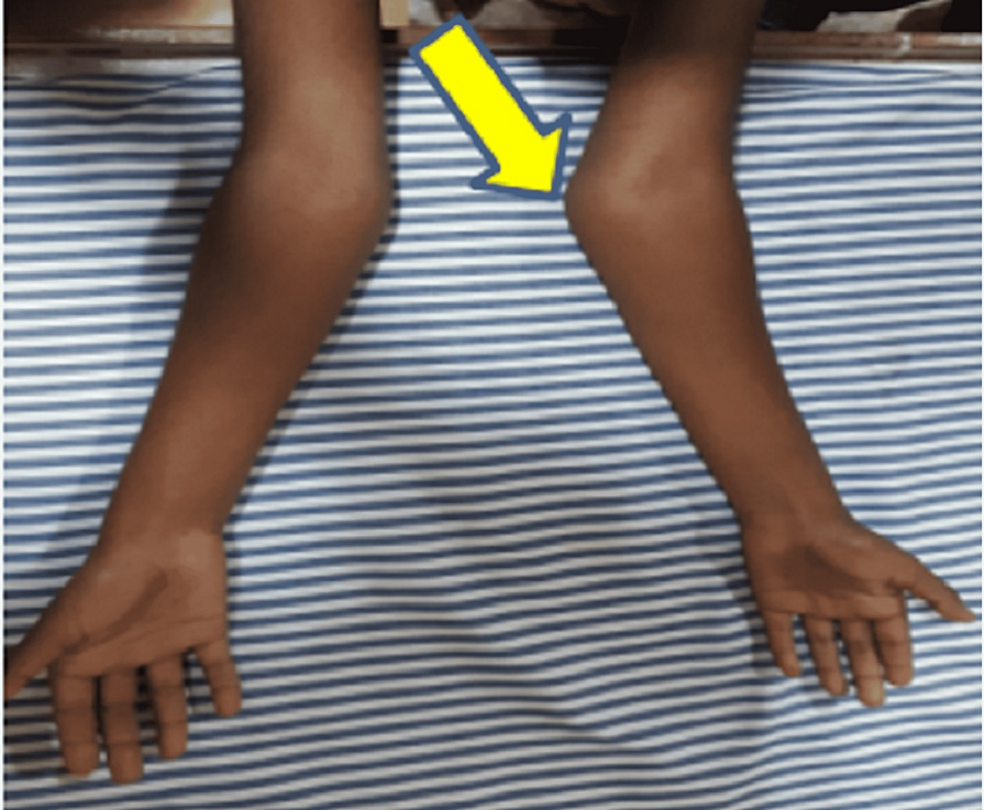
Bilateral carrying angle more in the left side

**Figure 5:**
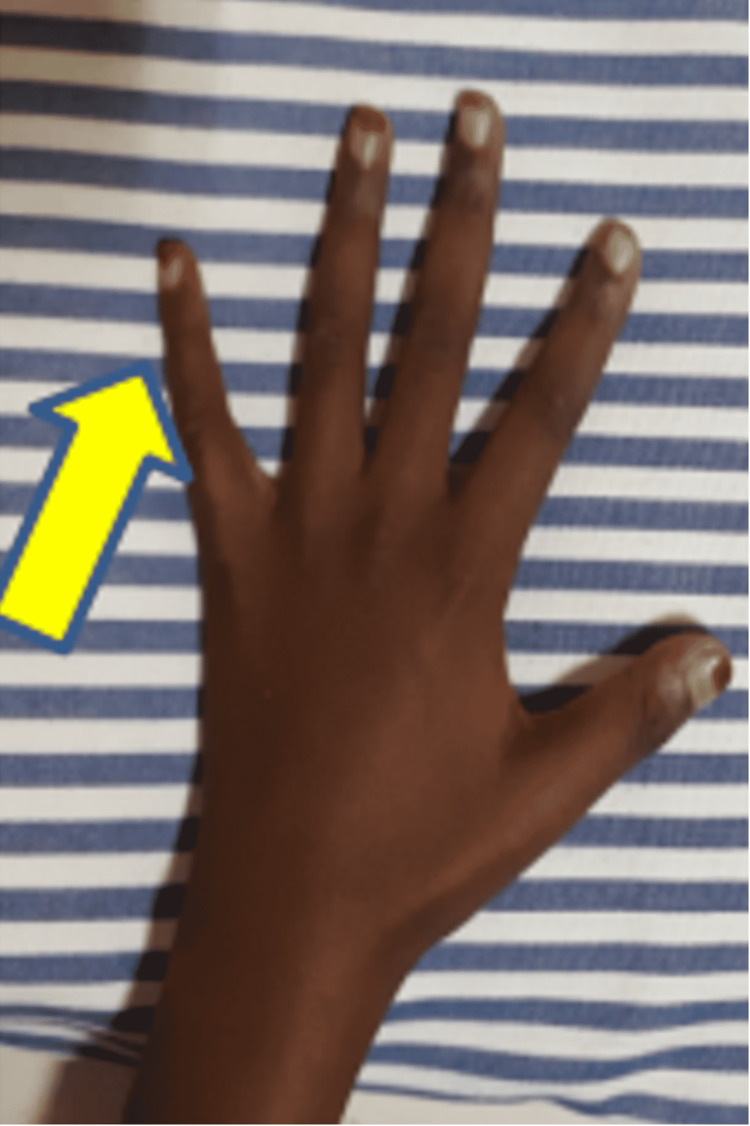
Ulnar clawing corrected over the left little finger

**Figure 6:**
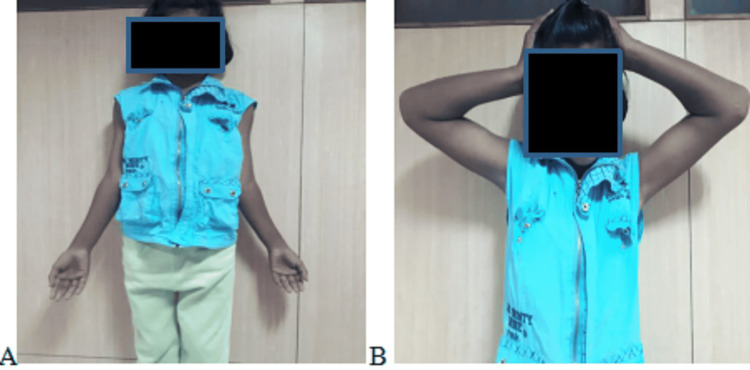
Clinical picture of (A) elbow extension and (B) elbow flexion showing successful functional range of motion after surgical management

Postoperatively, the patient received intravenous amoxicillin-potassium clavulanate twice daily for seven days, followed by oral amoxicillin-potassium clavulanate twice daily for seven days (dosage was based on the weight of the patient). A postoperative radiograph of the operated elbow joint is shown in Figure 2. The three-month follow-up plain radiograph of the left elbow joint is shown in Figure 3. The bilateral carrying angle was more on the left side, as shown in Figure 4. Ulnar nerve clawing over the left little finger was corrected, as shown in Figure 5. Ulnar nerve injury recovered on its own, possibly neuropraxia. During the last follow-up, six months post-injury, the patient had recovered from ulnar claw hand and had gained a successful full range of motion over her left elbow joint, as shown in Figure 6.

## Discussion

Flexion-type accounts for a minority of all supracondylar fractures of the humerus, and they warrant special attention. This is due to the high rate of necessity for open reduction and their potential for ulnar nerve injury or entrapment. Non-displaced or minimally displaced flexion-type supra-condylar fractures of the humerus can be treated with long arm casting. Displaced flexion-type supra-condylar humerus fractures require surgical reduction and stabilization [4]. 6.5% to 19% of cases of displaced fractures present with neural injuries, and such injuries are uncommon in non-displaced fractures [5].

Injuries can be either primary lesions (appearing before surgery) or secondary lesions (appearing after reduction and fixation of the fracture). Stretching, entrapment, or disruption of the nerve can happen during primary lesions caused by fracture displacement. Immobilization during hyperflexion, iatrogenic injuries by fixation, or excessive manipulation may lead to secondary lesions [6,7]. Pinning errors were significantly more frequent in surgeries performed at night [8]. Delniotos et al. in their study proposed that orthopedic surgeons should be aware, and family members of those patients should be informed, that the likelihood of an open reduction in flexion-type supracondylar fracture is extremely high. Open reduction is needed not only to achieve an anatomic reduction of the fracture but to make sure that the ulnar nerve is not entrapped between the proximal and distal fragment [9]. 

Valle-Hernandez et al., in their study, noted that the neurological complication rates were 10% (33 patients). Neurologic complications after displacement of the distal fragment were 13.5% for posteromedial displacement and 11.8% for postero-lateral displacement. The female-to-male ratio, fracture type, and complications (e.g., infection, vascular and neurologic) were similar to those reported in the literature [10]. Green et al., in their article, demonstrated that crossed-pin fixation can be performed safely and reliably and is an appropriate treatment option for unstable supracondylar fractures of the humerus [11].

## Conclusions

The patient in our case report with delayed presentation had a successful clinical outcome and functional range of motion after Kirschner's wire fixation. Successfully treating the flexion-type supracondylar fractures and neurological deficit is a challenge. Patients and families should be counseled regarding the high rate of open reduction for flexion-type supracondylar fractures of the humerus, particularly those with an associated ulnar nerve injury.
